# Water and war: The effect of functioning chlorinated water stations in reducing waterborne diseases during conflict in northwest Syria, 2017–2021

**DOI:** 10.1371/journal.pgph.0002696

**Published:** 2023-12-27

**Authors:** Rohini Haar, Sarah Abdelrahman, Evan Muzzall, Ibrahim Aladhan, Kasim Shobak, Mohamad Rami Kawas, Aya Aksh, Alden Hooper Blair, Arthur Reingold, Naser AlMhawish

**Affiliations:** 1 Division of Epidemiology, UC Berkeley School of Public Health, Berkeley, California, United States of America; 2 Evan Muzzall, Stanford Libraries, Stanford University, Palo Alto, California, United States of America; 3 Assistance Coordination Unit, Gaziantep, Turkey; 4 Institute for Global Health Sciences, University of California, San Francisco, California, United States of America; 5 Syria Public Health Network, London, United Kingdom; Indian Institute of Public Health Shillong, INDIA

## Abstract

Since 2011, Syria has been engulfed in a complex conflict marked by both targeted and indiscriminate attacks on civilians and civilian infrastructure. Water infrastructure has been continuously targeted, exacerbating problems with contamination of and access to clean adequate drinking water, and increasing the risk of waterborne diseases. We aimed to determine whether having access to more functional and chlorinated water stations is associated with a reduced risk of waterborne disease in northwest Syria. We examined the effect of functioning chlorinated water stations on the incidence of waterborne disease in 10 districts of Northwest Syria between January 1, 2017, and June 30, 2021, using weekly reported disease surveillance data and data from a water, sanitation, and hygiene (WASH) system evaluation program of the Assistance Coordination Unit (ACU). We ran eight negative binomial models to examine the association between functioning chlorinated water stations and the incidence of four of the five waterborne diseases: acute bloody diarrhea (ABD), acute other diarrhea (AOD), acute jaundice syndrome (AJS), and severe typhoid fever (STF). Dose-response models were used to investigate how the incidence of disease can theoretically be reduced as functioning and chlorinated water stations strategically increase. Compared to areas with lower quintiles of functioning and chlorinated water stations, the rates of the four waterborne diseases were lower in areas with higher quintiles of functioning and chlorinated water stations. Exposure to functioning water stations had a stronger association with lower rates of waterborne diseases than exposure to chlorinated water stations. Dose-response models demonstrate a potential for curbing the incidence of acute diarrhea and acute jaundice syndrome. The results of this study provide an understanding of the effects of water station functionality and chlorination in conflict settings. These findings support greater prioritization of WASH activities in countries experiencing violence against civilian infrastructure.

## I. Background

In 2019, unsafe drinking water and poor sanitation and hygiene were estimated to have caused the deaths of nearly 1 million people globally, including an estimated 300,000 children under the age of five [1]. The risk of illness related to inadequate water, sanitation, and hygiene (WASH) is much higher in humanitarian emergency settings, such as conflicts and natural disasters. WASH conditions are known to be an important determinant of diarrheal deaths in humanitarian emergencies, accounting for 40 percent of such deaths [1]^.^ Functional WASH systems are vital in preventing displacement, reducing the risk of outbreaks, and reducing malnutrition [2]. WASH interventions that are sometimes implemented in emergency responses include programs to increase drinking water access, quality and quantity, isolate feces and other contaminants from drinking water sources, and promote good hygiene practices at individual, household and community levels. The repair or construction of water infrastructure such as water treatment plants and sewage systems as well as support for water facilities are much needed but often lacking interventions [3, 4]. Conflict-affected populations are at increased risk of numerous poor health outcomes, including waterborne diseases, as a result of insufficient access to WASH facilities and resources and numerous other factors [5–7].

Syria has been the site of a complex conflict marked by both targeted and indiscriminate attacks on civilians and civilian infrastructure since 2011 [8]. The ongoing conflict has resulted in an estimated 6.6 million Syrian people forced to flee the country as refugees, an additional 6.7 million Syrian people displaced internally, and over 12 million more Syrian people requiring humanitarian/emergency WASH services in the country [9–12]. Water infrastructure has been frequently targeted during the conflict, exacerbating existing deficiencies and further reducing access to adequate clean drinking water for the population in affected areas [5, 13]. The functionality of the water infrastructure in Syria has declined significantly during the conflict because of a shortage of electricity; limited financial resources and restricted equipment resulting from international sanctions; and the outflow of trained staff due to displacement [9]. As a result, two thirds of the Syrian population do not have regular access to clean drinking water as a result of contamination of water sources caused by the destruction of infrastructure and floods, including leakage of sewage into the water system (known as wastewater floods) [13]. Regulating access to clean water has also been leveraged by all parties in the conflict to control civilian populations and to pressure opponents [14, 15]. And ongoing violence against healthcare has resulted in reductions in the health workforce required to care for ill patients [11, 16].

Due to a complex interplay of problems, including security and financial restrictions during conflict and war, understaffing, the lack of availability of accurate data, and complex operational conditions, there have been few studies examining the effect of WASH projects on health in conflict settings. A recent systematic review found that there was a lack of high-quality information and of data concerning the effectiveness of WASH interventions in conflict settings [6], and studies in Syria, perhaps the most impacted State in recent years, have been scarce. One study analyzed cross-sectional household survey data from southern Syria in 2016–2017 and found that while access to piped water dropped and households spent about 20% of their income on water, sanitation and hygiene access were protective against childhood diarrhea [17]. A recent literature review and analysis of EWARN (Early Warning Alert and Response Network) data on five waterborne diseases in Northeast Syria demonstrated the deliberate targeting of water infrastructure and the need for more granular analysis of such attacks and disease to assess the health impacts as their district-level analysis yielded concerning trends [15, 18].

To fill this gap, we investigated the functionality and chlorination of water stations and the associated rates of waterborne diseases during the conflict in northwest Syria. We chose Northwest Syria as our study site because of the intensity of the conflict there as well as the availability of robust data that the Assistance Coordination Unit (ACU), a local NGO working on information management, surveillance and capacity building around health, water, nutrition and other related issues in opposition-controlled Syria, has collected in this region [19]. The ACU has estimated that 75% of the population in northwest Syrian communities, and 42% of people in internally-displaced camps use water from the water stations that they manage (63% of the total population of their catchment area), making this a critical component of northwest Syria’s WASH system [20]. We analyzed weekly reported disease surveillance reports and data from a WASH system evaluation conducted by ACU’s EWARN program, in an effort to provide a better understanding of the effect of water station functionality and chlorination in conflict settings and provide more granular evidence in support of improving and expanding WASH activities in countries experiencing conflict and war. Dose-response models were included to assess the potential for reductions in incidence of waterborne diseases.

## II. Methods

### Ethics statement

This study was reviewed and considered exempt by the Committee for the Protection of Human Subjects of the University of California, Berkeley. We also discussed the project with ACU staff and other colleagues in Syria to ensure local ethical and security concerns and factors were taken into account. We conducted this study via a collaborative partnership with ACU staff, Syrian diaspora researchers and technical experts.

We examined, at the ecologic level, the effect of functioning and chlorinated water stations on the incidence of waterborne disease in 10 districts in northwest Syria between January 1, 2017, and June 30, 2021.

### Study population

**Fig 1:**
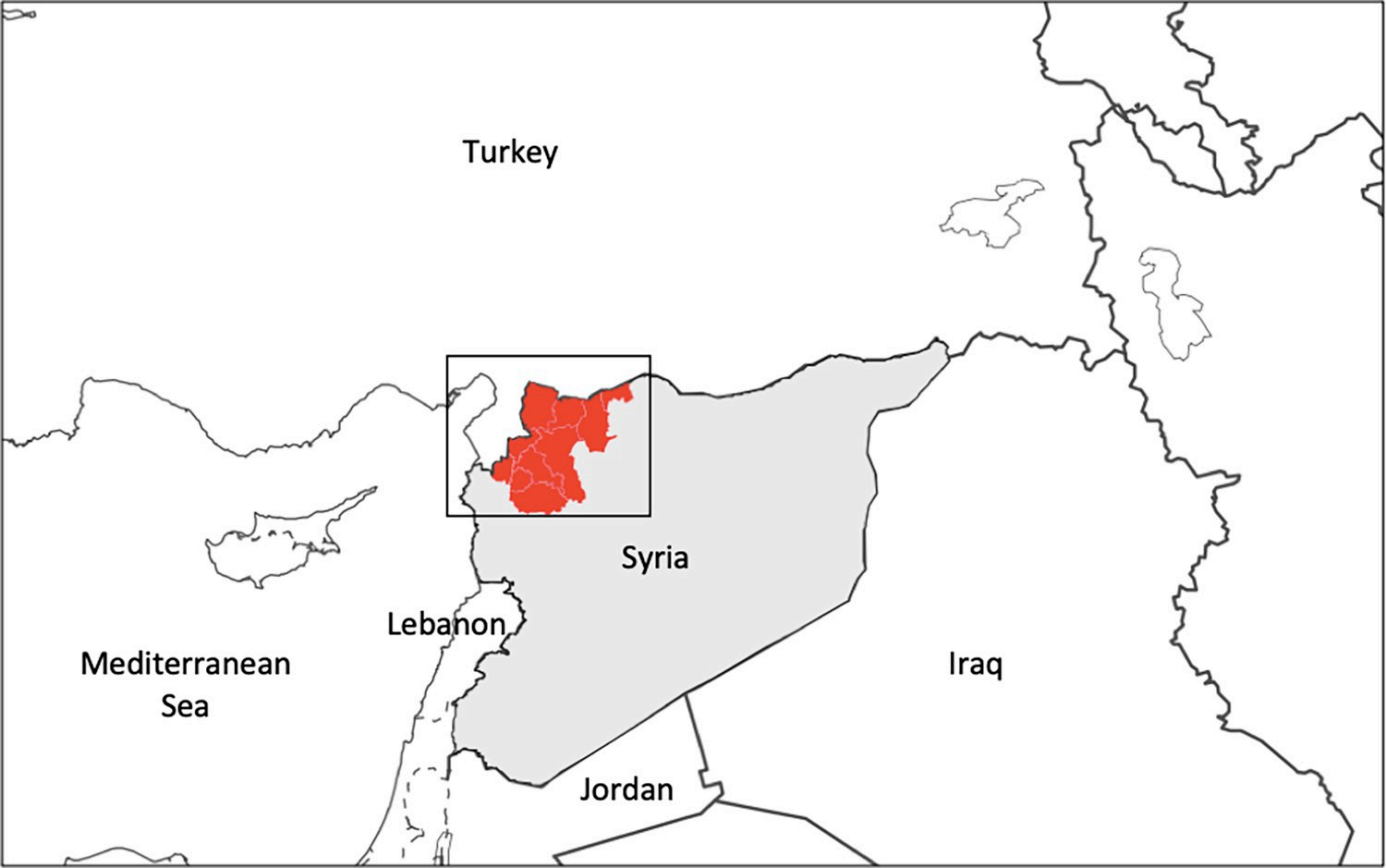
Northwest Syria and its borders.

The population of interest resides in northwest Syria, which consists of the area accessible from Gaziantep, Turkey and not under Syrian government-control. Northwest Syria was chosen as the area for granular analysis because a) conflict intensity in 2017 through 2021 was concentrated in this area [21], b) water station and disease data were consistently available, and c) colleagues were already analyzing other northeast Syrian data (albeit without water station data). Our area of study included five districts in the Idleb governorate: Ariha, Harem, Idleb, Al Ma’ra, and Jisr-Ash-Shugur, as well as five districts in the Aleppo governorate: Jebel Saman, Afrin, A’zaz, Al Bab, and Jarablus (Fig 1).

### Data collection

#### Waterborne disease data

Data on cases of waterborne diseases were collected by ACU’s surveillance system, called EWARN [22]. EWARN is an active surveillance program in which surveillance data are requested from healthcare providers in health facilities in Syria based on a set criteria (Table 1) [22]. EWARN collects data on 13 diseases and conditions, selected for their potential to cause epidemics, their association with high morbidity and mortality, and the potential for intervention in Syria [22]. Of the 13 diseases, five were considered waterborne diseases for the purposes of this analysis: acute watery diarrhea (AWD), acute bloody diarrhea (ABD), acute other diarrhea (AOD), acute jaundice syndrome (AJS), and severe typhoid fever (STF). AOD reflects the total number of all diarrhea diseases other than AWD and ABD. In practical terms, AWD frequently stands in for cholera and similar diseases while ABD stands in for Shigellosis.

**Table 1:** Case-definitions for each of the five waterborne diseases [22].

*Disease*	ACU Case Definition
*Acute watery diarrhea*	any person aged five years or more with severe dehydration or death from acute watery diarrhea in the past 24 hours, with or without vomiting, where cholera is suspected.
*Acute bloody diarrhea*	acute diarrhea (i.e., three or more abnormally loose or fluid stools in the past 24 hours) with visible blood in stool, when shigellosis is suspected.
*Acute another diarrhea*	acute diarrhea (i.e., three or more loose stools in the past 24 hours), not due to bloody diarrhea or suspected cholera.
*Acute jaundice syndrome*	the onset of jaundice (i.e., yellowing of the sclera of the eyes or of the skin or dark urine); severe illness, including signs of fatigue, nausea, vomiting and abdominal pain; and the absence of any known precipitating factors, when hepatitis A or hepatitis E is suspected.
*Suspected severe typhoid fever*	any person with acute illness and fever of at least 38°C for three or more days with abdominal symptoms (i.e., diarrhea or constipation or abdominal tenderness progressing to prostration) and relative bradycardia or a symptomatic case contact of someone with a confirmed case of typhoid.

As a syndromic infectious disease surveillance system, EWARN reports on a weekly basis the number of clinically suspected cases of each disease. Suspected cases are not all laboratory-confirmed, due to many constraints upon facilities operating in conflict settings, and are instead reported if they meet clinical case definitions [22, 23]. This is standard procedure in emergency settings, as clinically suspected cases are the measure most heavily relied upon by humanitarian response organizations and reported by the WHO when widespread and consistent laboratory testing is not available [22, 23].

We received data from the time period between January 1, 2017, and June 30, 2021, as aggregated weekly case counts of each of the five waterborne diseases. No personally identifiable data were included, and reporting at the district level (rather than community level) was chosen to avoid the potential for case-tracing and to protect the identities of communities at risk. Data are available to the public via ACU’s dashboard [24].

#### Water station data

Data on functionality and chlorination status of water stations were collected by ACU’s Water and Environmental Sanitation program between January 1, 2017, and June 30, 2021 [25]. This program is a part of ACU’s EWARN system and provides support for water quality testing, procedures for obtaining safe drinking water and improving sanitation services, and promoting hygiene. Functionality and chlorination status of water stations were reported biweekly by WASH officers on the ground in northwest Syria and were aggregated at the district level. We aggregated the data at the district level rather than at the water station level because there is some inconsistency between the sub-district where the water stations are located and the sub-districts where the populations served reside.

Functionality status was reported as either functioning or non-functioning. A functioning water station denotes that water is actively processed from this station and distributed to districts in the community that week. A non-functioning water station is not processing nor distributing water that week. ACU records functionality among a range of water station types including boosting stations, shallow and regular well stations, spring stations and water treatment stations [26]. Chlorination status of a water station was reported as either chlorinated or not chlorinated. If a water station is functioning, then it can either be chlorinated or not chlorinated (i.e., if a water station is not functioning, then it cannot be chlorinated). Chlorination status was measured by taking a sample of water at each station and measuring if the percentage of free chlorine residual in the sample was adequate [25]. ACU uses a cut-off value of 0.1ppm of free residual chlorine to classify a water station as either chlorinated or not-chlorinated. This was done biweekly by WASH officers using a portable chlorine sensing device [27]. In emergencies and war zones, portable sensors are an appropriate means of determining the quality of water because they provide the possibility of running tests on the ground, are easy to transport, and testing can be performed without electrical power [25].

Population estimates for Syria between 2017 and 2021 were obtained from the Humanitarian Needs Assessment Programme (HNAP) of the United Nations Office for the Coordination of Humanitarian Affairs (UNOCHA) [28, 29]. UNOCHA reported population estimates monthly by collecting data using questionnaires and interviews, consulting key informants, and making observations about population movement. Population estimates were aggregated at the district level. Visualizing the monthly population estimates, we found that they rose or dropped incrementally over time without dramatic fluctuations month to month. To match our weekly disease data, we applied these monthly population estimates as weekly population estimates (the same monthly estimate for each of the weeks within the month) to calculate the weekly incidence of each of the waterborne diseases. Of note, data collection was suspended for the period between December 2017 to April 2018 for conflict-related conditions; as a result, we did not have population estimates for that time period and removed these observations from the analysis.

### Statistical analyses

The two exposure variables were converted from binary categorical variables (functioning vs non-functioning and chlorinated vs non-chlorinated water stations in each district) to continuous variables (the percent of functioning water stations and the percent of chlorinated water stations per district) because there were multiple water stations per district. These two continuous exposure variables of functionality and chlorination percentages were then converted into quintiles to account for the non-linearity between the exposures and the outcome and to allow a more tangible interpretation of our results [30]. The outcome variables included four of the five suspected waterborne disease case counts. We excluded acute watery diarrhea (AWD) as an outcome because there were too few cases reported (n = 11 cases) and therefore, we lacked sufficient power to detect meaningful differences.

The total case counts of suspected waterborne diseases during the study period were over-dispersed (i.e., there was more variability than expected relative to a Poisson distribution, which is a common approach to analyze ecological count data), where the mean does not equal the variance. This is problematic because the true standard deviation of the exposure variable is underestimated, *and* the significance of the independent variables will be overstated by the model. We thus chose a negative binomial regression model to account for latent heterogeneity [31]. We ran a total of eight models: for the first four models, the predictor variable for each of the diseases was a five-level categorical variable of percent of functioning water stations (Eq 1) and for the other four models, the predictor variable was a five-level categorical variable of percent of chlorinated water stations (Eq 2). In each model, the outcome was one of the four waterborne diseases (ABD, AOD, AJS, and STF). All models were adjusted for district and week and included an offset term of the total population and a continuous interaction term between percent of functioning and percent of chlorinated water stations. These adjustments were made based on empirical evidence and theoretical importance to understanding the underlying relationship between the primary exposures and outcomes [15, 17, 32]. To account for an incubation period of disease, the outcome variable Severe Typhoid Fever (STF) was lagged by two weeks and the outcome variable Acute Jaundice Syndrome (AJS) was lagged by six weeks, while Acute Bloody Diarrhea (ABD) and Acute Other Diarrhea (AOD) were not lagged based on previous studies [33–36]. Our estimates included incidence rate ratios for each of the four waterborne diseases. A 95% confidence interval that did not cross the null value of one was considered statistically significant [37]. Finally, a Vuong’s Test, a non-nested likelihood ratio test, was done to compare our negative binomial regression models to a simple Poisson model to determine which model was a better fit. Statistical analyses and tests were conducted in R (version X.Y.Y; R Core Team), while ArcGIS [38] was used for creating visual maps.

Data on case counts of each waterborne disease were also reported and split into male cases and female cases and into cases <5 years of age and cases ≥5 years of age for the descriptive analysis and the dose response analysis.

Eq 1. Negative binomial regression model for the association between case counts of each suspected waterborne disease (i.e., AOD, ABD, STF, and AJS) and the percent of functioning water stations in quintiles:

$$log(E[x,d,t,p)=x_{1}\beta_{1}+x_{2}\beta_{2}+x_{3}\beta_{3}+x_{4}\beta_{4}+x_{5}\beta_{5}+d\beta_{6}+t\beta_{7}+y\beta_{8}+log(p)$$

where:

- *x*_1_ is the indicator for percent of functioning between 0 to <34.5 (reference group)
- *x*_2_ is the indicator for percent of functioning between 34.5 to <52.5
- *x*_3_ is the indicator for percent of functioning between 52.5 to <77.4
- *x*_4_ is the indicator for percent of functioning between 77.4 to <86
- *x*_5_ is the indicator for percent of functioning between 86 to 100
- *d* is 10-level categorical district
- *t* is continuous time in weeks
- *y* is the interaction term between percent of functioning and percent of chlorinated
- p is offset term of log of total population

Eq 2. Negative binomial regression model for the association between case counts of each suspected waterborne disease (i.e., AOD, ABD, STF, and AJS) and the percent of chlorinated water stations in quintiles:

$$log(E[x,d,t,p)=x_{1}\beta_{1}+x_{2}\beta_{2}+x_{3}\beta_{3}+x_{4}\beta_{4}+x_{5}\beta_{5}+d\beta_{6}+t\beta_{7}+y\beta_{8}+log(p)$$

where:

- *x*_1_ is the indicator for percent of chlorinated between 0 to <33.3 (reference group)
- *x*_2_ is the indicator for percent of chlorinated between 33.3 to <55.8
- *x*_3_ is the indicator for percent of chlorinated between 55.8 to <75.0
- *x*_4_ is the indicator for percent of chlorinated between 75.0 to <88.2
- *x*_5_ is the indicator for percent of chlorinated between 88.2 to 100
- *d* is 10-level categorical district
- *t* is continuous time in weeks
- *y* is the interaction term between percent of functioning and percent of chlorinated
- p is offset term of log of total population

Dose-response models were used to investigate whether incidences of the five diseases can be reduced by increasing the percentage chlorinated water stations.

Dose-response models were fitted using the Dose Response Curve (drc) R package to predict a response, given a specified dose, to estimate the dose amount (number of functioning or chlorinated water stations) required to reduce the response (incidences of each of the diseases) [39, 40]. The Median Effective Dose (ED50) for a given dose is the point along a dose-response curve when the log dose along the x-axis intersects at the point on the curve to indicate 50% of the desired response on the y-axis. This point specifies the required dose to produce 50% of the desired response in 50% of the population.

A total of 30 three parameter log-logistic models were used with parameters slope, upper limit, and offset. The lower limit was fixed to zero because there are instances of zero incidence of the disease; that is, not every area reported a case of disease. Response variables were the number of cases of the five diseases by: total cases, age group 0–4 years, age group > = 5 years, males only, and females only. Doses were percent functioning water stations and percent chlorinated water stations for acute other diarrhea and percent functioning water stations (with two-week lag period) and percent chlorinated water stations (with two week and six week lag periods) to account for the incubation periods of severe typhoid fever and acute jaundice syndrome, respectively.

## III. Results

### Descriptive analysis

**Fig 2:**
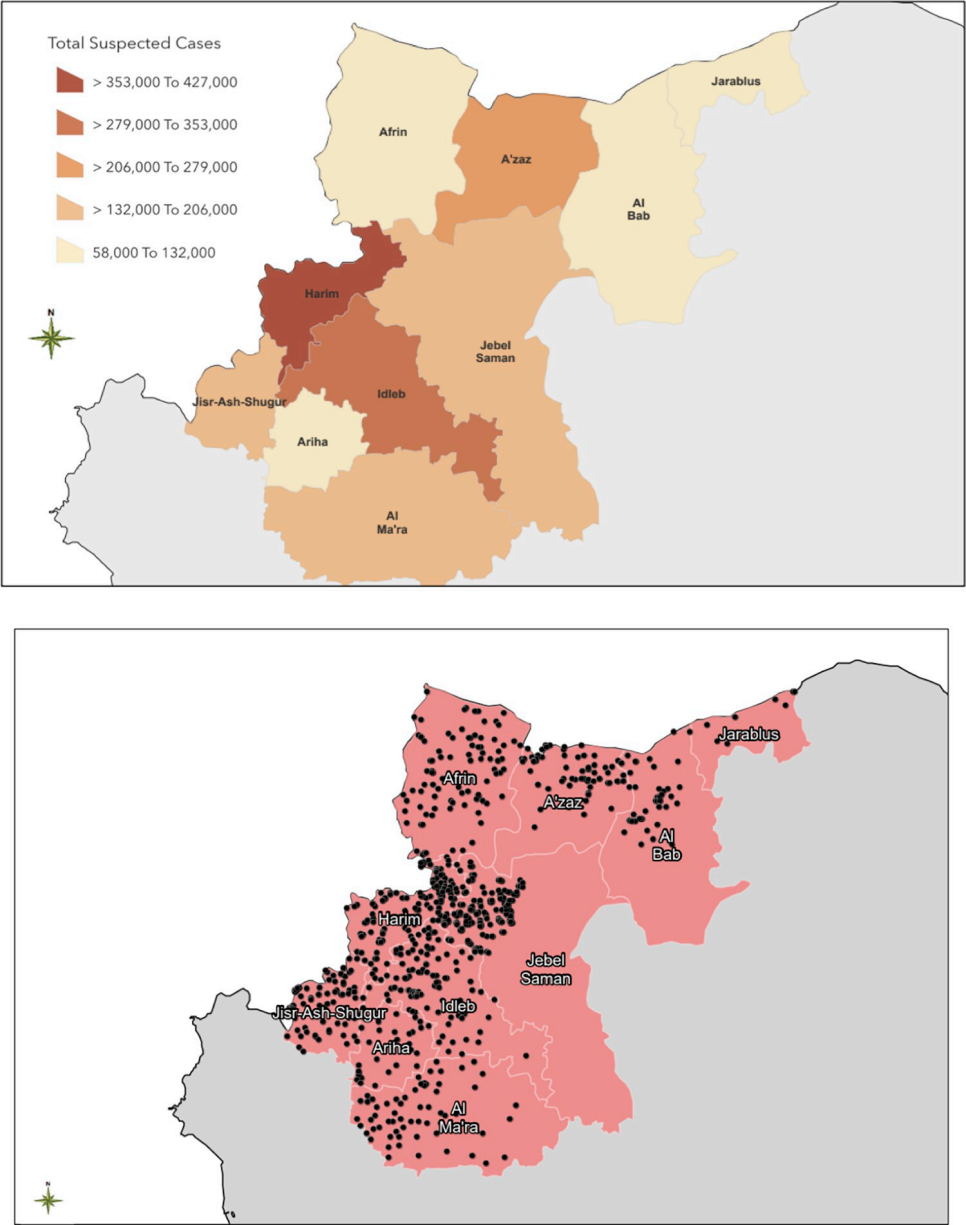
Total number of suspected cases of waterborne diseases and distribution of water stations in northwest Syria by district: January 2017 –June 2021.

A total of 1,834,678 suspected cases of the five waterborne diseases were reported within the 10 districts in northwest Syria during the 4.5-year study period. Jarablus district had the lowest number of total suspected waterborne disease cases during the study period, ranging from one to 958 weekly cases (median = 229), while Harim had the highest number of total suspected cases, ranging from 556 to 4251 weekly cases(median = 1728) (Fig 2).

**Fig 3:**
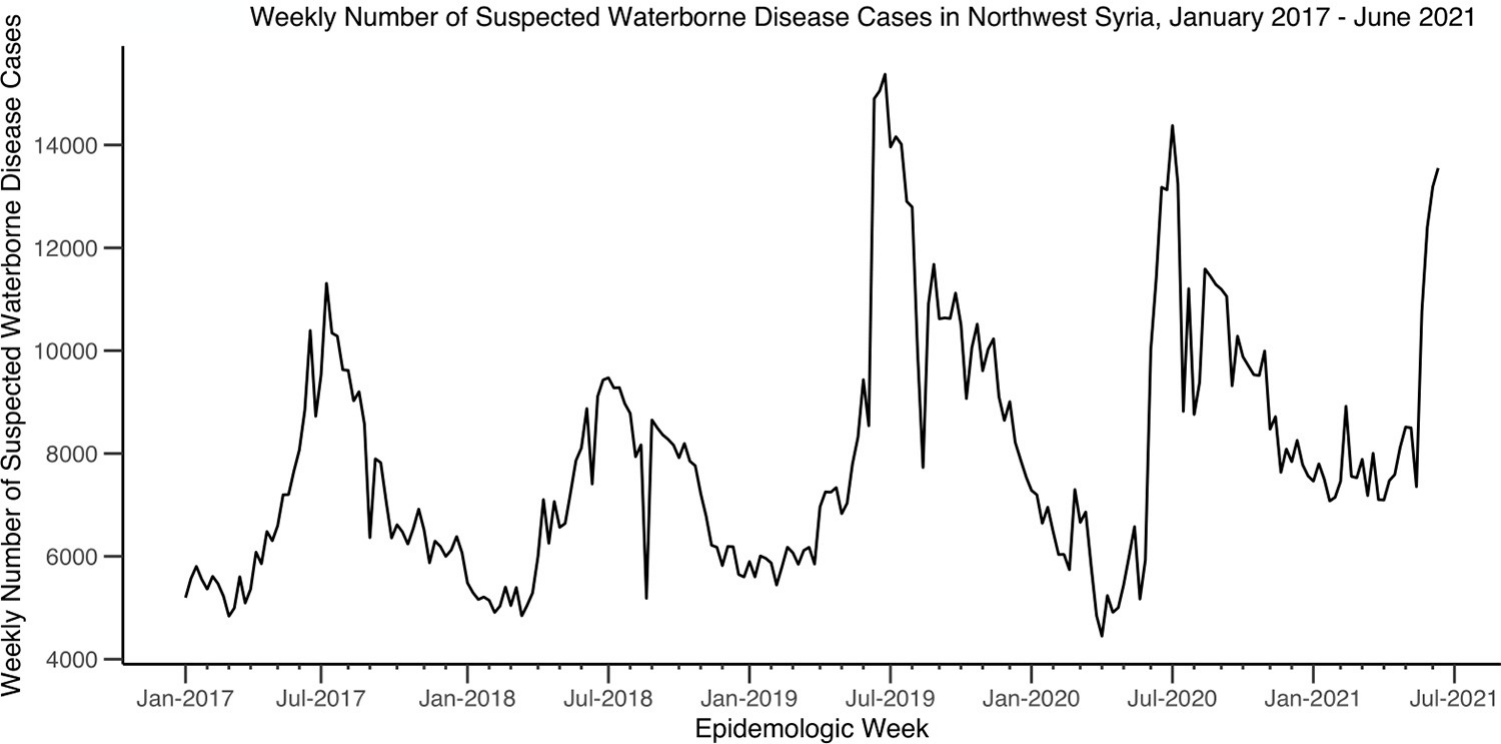
Weekly number of suspected waterborne disease cases in northwest Syria, January 2017 –June 2021.

**Fig 4:**
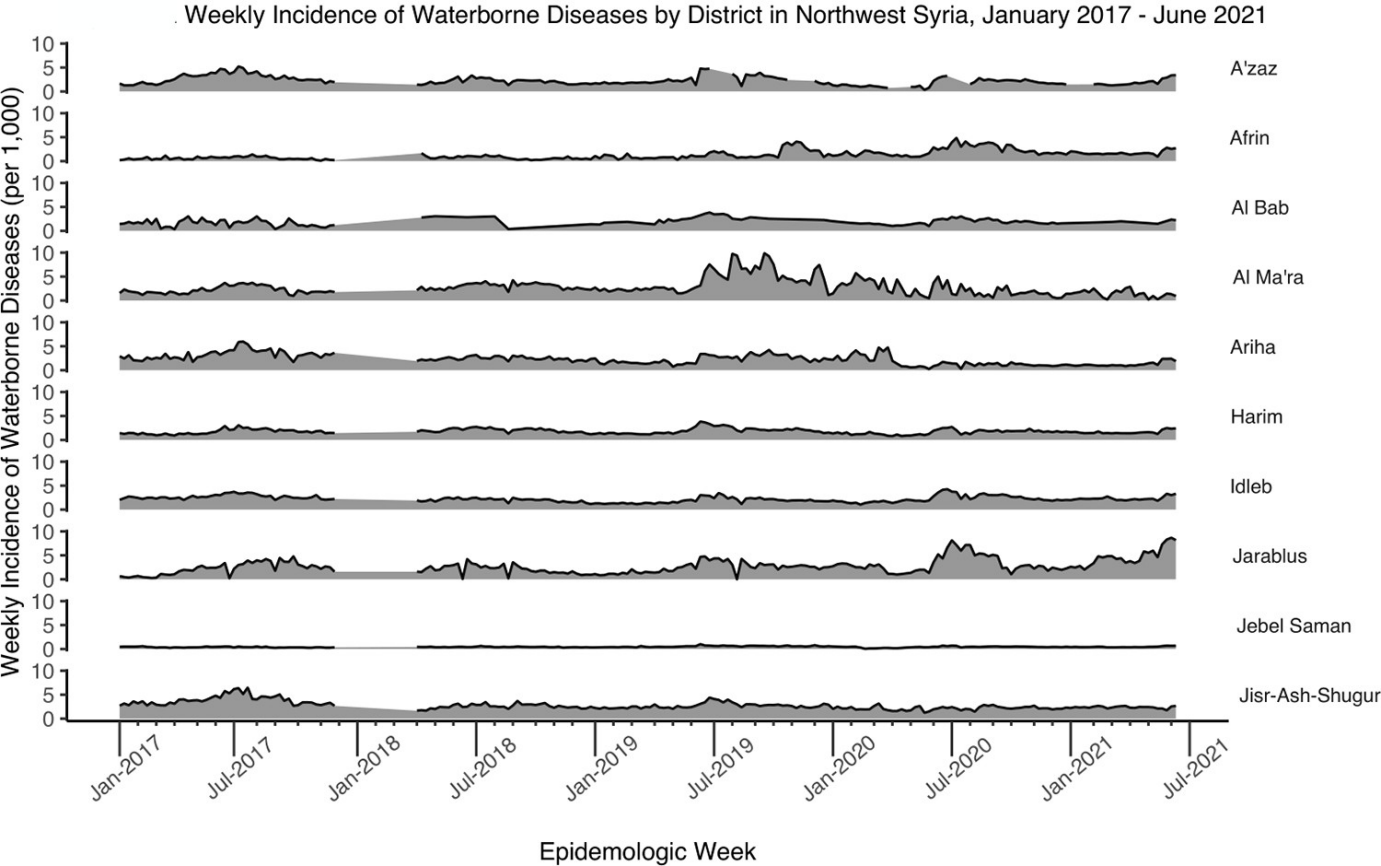
Weekly incidence of waterborne diseases by district in northwest Syria, January 2017 –June 2021. *Note: population estimates were not available from December 2017 to April 2018.

Consistent with having the lowest population size (median = 88,334 people), Jarablus had the lowest number of cases, but it had the highest incidence rate (median = 252 per 100,000, ranging from 1 to 865 per 100,000) (Tables 2 and 3). Jebel Saman had the highest population size over the 4.5 year study period (median = 1,972,589) but had lowest incidence rate (median = 42 per 100,000, ranging from 4 to 99 per 100,000) during the study period (Tables 2 and 3). The total annual numbers of reported cases of waterborne diseases were similar each year, ranging from 360,000 to 470,000. In all the districts, the numbers of cases of suspected waterborne diseases were highest in the summer months (June to August) each year (Figs 3 and 4).

**Table 2:** Suspected cases of waterborne diseases by year, northwest Syria, January 2017 –June 2021.

Governorate	District	2017 n (%)	2018 n (%)	2019 n (%)	2020 n (%)	2021 n (%)	Total Cases n (%)
Aleppo		118838 (33)	109527 (30)	169492 (36)	184269 (42)	81651 (40)	663,777 (36)
	Afrin	9227 (3)	5940 (2)	17025 (4)	47479 (11)	18649 (9)	98320 (5)
	Al Bab	10995 (3)	5337 (1)	14583 (3)	24051 (6)	3969 (2)	58935 (3)
	A’zaz	51582 (14)	48185 (13)	66433 (14)	57387 (13)	26944 (13)	250531 (14)
	Jarablus	9415 (3)	8684 (2)	11,402 (2)	16822 (4)	12309 (6)	58632 (3)
	Jebel Saman	37619 (10)	41381 (11)	60049 (13)	38530 (9)	19780 (10)	197359 (11)
Idleb		245509 (67)	251977 (70)	300930 (64)	250966 (58)	121519 (60)	1170901 (64)
	Al Ma’ra	51643 (14)	56626 (16)	51384 (11)	1535 (0)	501 (0)	161689 (9)
	Ariha	30420 (8)	27940 (8)	31651 (7)	11391 (3)	5417 (3)	106819 (6)
	Harim	52837 (15)	75583 (21)	110,717 (24)	127,080 (29)	60255 (30)	426472 (23)
	Idleb	69282 (19)	59210 (16)	69,530 (15)	76,919 (18)	37992 (19)	312933 (17)
	Jisr-Ash-Shugur	41327 (11)	32618 (9)	37648 (8)	34041 (8)	17354 (9)	162988 (9)
**Total:**		364,347 (20)	361,504 (20)	470,422 (26)	435,235 (24)	203,170 (11)	1834678 (100)

**Table 3:** Weekly median of water stations, including functionality and chlorination status; of total waterborne disease cases; of incidence; and of total population; by district, northwest Syria, January 2017 –June 2021.

Governorate	District	Water Stations median (min, max)	Functioning Stations median (min, max)	Chlorinated Stations median (min, max)	Total Waterborne Disease Cases median (min, max)	Incidence (per 100,000) median (min, max)	Total Population median (min, max)
Aleppo							
	Afrin	64 (14, 83))	20 (1, 55))	2 (0, 34))	247 (33, 2069))	101 (11, 484))	308022 (136132, 458234))
	Al Bab	9 (1, 24))	9 (0, 19))	6 (0, 16))	402 (40, 957))	183 (33, 381))	233249 (61666, 344036))
	A’zaz	37 (15, 58))	31 (10, 51))	19 (0, 45))	985 (215,2394))	210 (33, 522))	497571 (345116, 701651))
	Jarablus	8 (7, 8))	5 (2, 8))	4 (1, 8))	229 (1, 958))	252 (1, 865))	88334 (73256, 111439))
	Jebel Saman	127 (64,150))	52 (8, 110))	8 (0, 40))	809 (66, 2123))	42 (4,991K))	1.972M(1736889, 2221016))
Idleb							
	Al Ma’ra	54 (3, 62))	38 (0, 53))	17 (0, 25))	763 (3, 2217))	238 (19, 986))	340459 (11622, 498881))
	Ariha	32 (25, 33))	10 (4, 17))	8 (2, 12))	458 (37, 1130))	222 (25, 596))	183688 (63589, 288626))
	Harim	50 (47, 144)))	44 (37, 95))	38 (33, 75))	1728 (556,4251))	168 (79, 383))	1.064M (597114, 1711036))
	Idleb	53 (34, 75))	41 (15, 44))	24 (10, 36))	1307 (643,2682))	214 (103, 425))	626480 (471920, 769943))
	Jisr-Ash-Shugur	40 (40, 96))	20 (15, 63)))	19 (11, 36))	667 (375, 1385))	251 (122, 647))	265781 (200058, 310351))

Reported as weekly totals and medians to match the disease data (with the minimum and maximum for the week), the largest number of water stations was in Harim district, ranging from 64 to 150 stations (median = 127), while Jarablus (median = 8) and Al Bab (median = 9) had the fewest water stations throughout the study period (Table 3). Jarablus, Al Bab, and Ariha districts had the smallest numbers of functioning stations, ranging from 0 to 19 per week, while Jebel Saman, Harim, and Idleb had the largest number of functioning stations, ranging from 8 to 110 per week (Table 4). Districts with the fewest chlorinated water stations were Afrin, Jarablus, Al Bab, and Ariha, ranging from 0 to 34 (median = 2, 4, 6, and 8, respectively). In contrast, Harim and Idleb districts had the largest number of chlorinated water stations, ranging from 10 to 75 (median = 38 and 24, respectively).

**Table 4:** Suspected cases of waterborne diseases by gender and age, northwest Syria, January 2017 –June 2021.

Governorate	District	Total Cases Male n (%)	Total Cases Female n (%)	Total Cases Ages 0–4 n (%)	Total Cases Ages 5+ n (%)	Total Cases n (%)
Aleppo		332709 (36)	331068 (36)	361595 (36)	302182 (36)	663,777 (36)
	Afrin	50305 (5)	48015 (5)	56245 (3)	42075 (6)	98320 (5)
	Al Bab	29288 (3)	29647 (3)	35702 (5)	23233 (4)	58935 (3)
	A’zaz	122709 (13)	127822 (14)	142837 (13)	107694 (14)	250531 (14)
	Jarablus	29885 (3)	28747 (3)	30119 (3)	28513 (3)	58632 (3)
	Jebel Saman	100522 (11)	96837 (11)	96692 (12)	100667 (10)	197359 (11)
Idleb		587732 (64)	583169 (64)	627128 (64)	543773 (64)	1170901 (64)
	Al Ma’ra	80824 (9)	80865 (9)	90101 (8)	71588 (8)	161689 (9)
	Ariha	54977 (6)	51842 (6)	57803 (6)	49016 (6)	106819 (6)
	Harim	211247 (23)	215225 (24)	228944 (23)	197528 (23)	426472 (23)
	Idleb	159743 (17)	153190 (17)	168567 (17)	144366 (17)	312933 (17)
	Jisr-Ash-Shugur	80941 (9)	82047 (9)	81713 (10)	81275 (10)	162988 (9)
**Total:**		920441 (50)	914237 (50)	988723 (54)	845955 (46)	1834678 (100)

During the study period, children less than five years of age accounted for over half (54%) of the cases of suspected waterborne diseases; suspected cases were evenly split between males and females (Table 4). Of the five waterborne diseases, Acute Other Diarrhea (AOD) accounted for the most cases (n = 1,608,102, 88%), while Acute Watery Diarrhea (AWD) accounted for the fewest reported cases (n = 11, 0%) (Table 5).

**Table 5:** Suspected cases of each waterborne disease by disease type, northwest Syria, January 2017 –June 2021.

Governorate	District	Total AOD^a^ Cases n (%)	Total ABD^a^ Cases n (%)	Total AWD^a^ Cases n (%)	Total AJS^a^ Cases n (%)	Total STF^a^ Cases n (%)	Total Cases n (%)
Aleppo:		575696 (36)	8576 (38)	1 (9)	31797 (37)	47707 (40)	663,777 (36)
	Afrin	83770 (5)	1291 (6)	0 (0)	6026 (7)	7233 (6)	98320 (5)
	Al Bab	50433 (3)	924 (4)	0 (0)	2683 (3)	4895 (4)	58935 (3)
	A’zaz	231037 (14)	1943 (9)	0 (0)	7469 (9)	10082 (8)	250531 (14)
	Jarablus	53630 (3)	328 (1)	0 (0)	2291 (3)	2383 (2)	58632 (3)
	Jebel Saman	156826 (10)	4090 (18)	1 (9)	13328 (16)	23114 (19)	197359 (11)
Idleb		1032406 (64)	13709 (62)	10 (91)	53173 (63)	71603 (60)	1170901 (64)
	Al Ma’ra	133355 (8)	3172 (14)	1 (9)	9324 (11)	15837 (13)	161689 (9)
	Ariha	91262 (6)	1347 (6)	6 (55)	5589 (7)	8615 (7)	106819 (6)
	Harim	383811 (24)	4236 (19)	1 (9)	16443 (19)	21981 (18)	426472 (23)
	Idleb	279481 (17)	3025 (14)	1 (9)	15057 (18)	15369 (13)	312933 (17)
	Jisr-Ash-Shugur	144497 (9)	1929 (9)	1 (9)	6760 (8)	9801 (8)	162988 (9)
**Total:**		1608102 (88)	22285 (1)	11 (0)	84970 (5)	119310 (6)	1834678 (100)

^a^Acute Other Diarrhea (AOD), Acute Bloody Diarrhea (ABD), Acute Jaundice Syndrome (AJS), Severe Typhoid Fever (STF)

**Fig 5:**
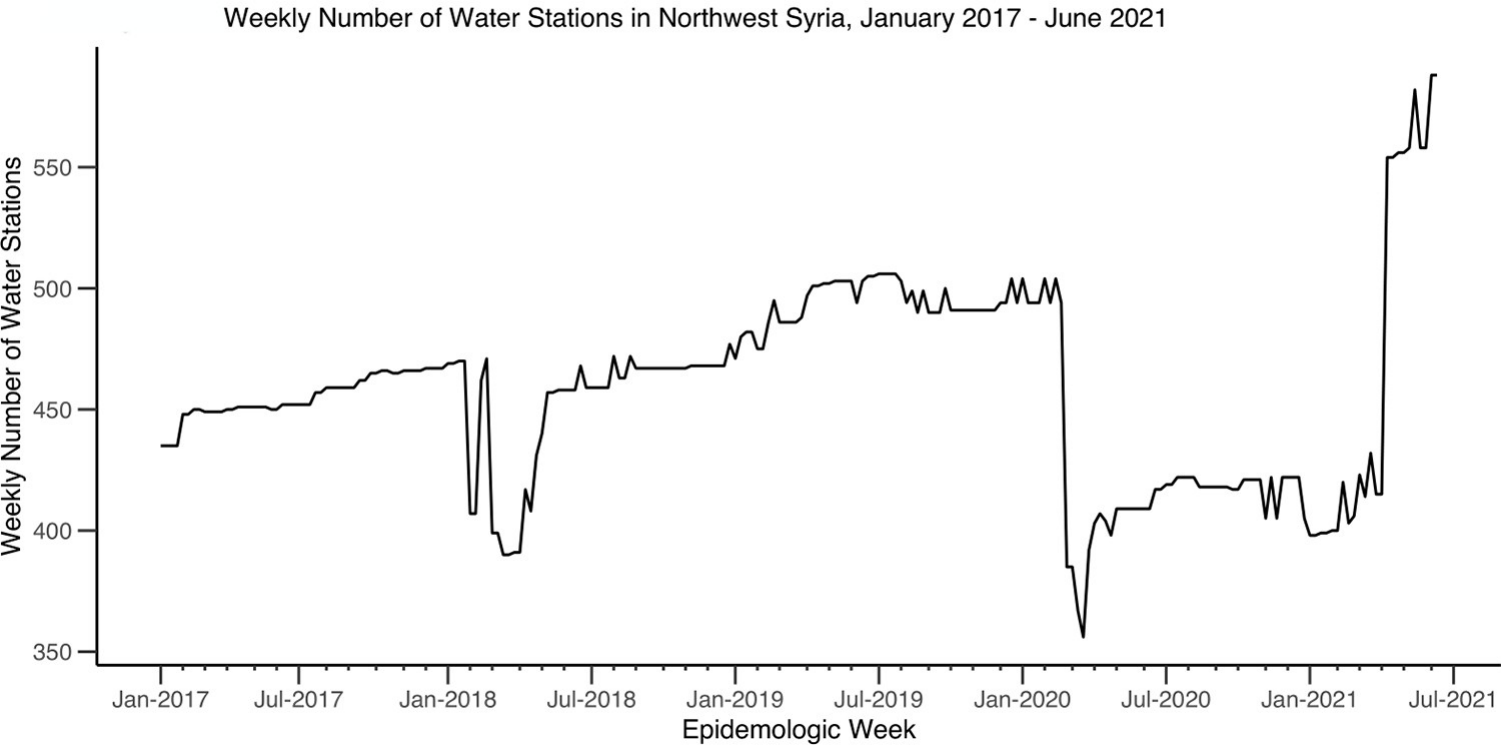
Weekly number of water stations in northwest Syria, January 2017- June 2021.

**Fig 6:**
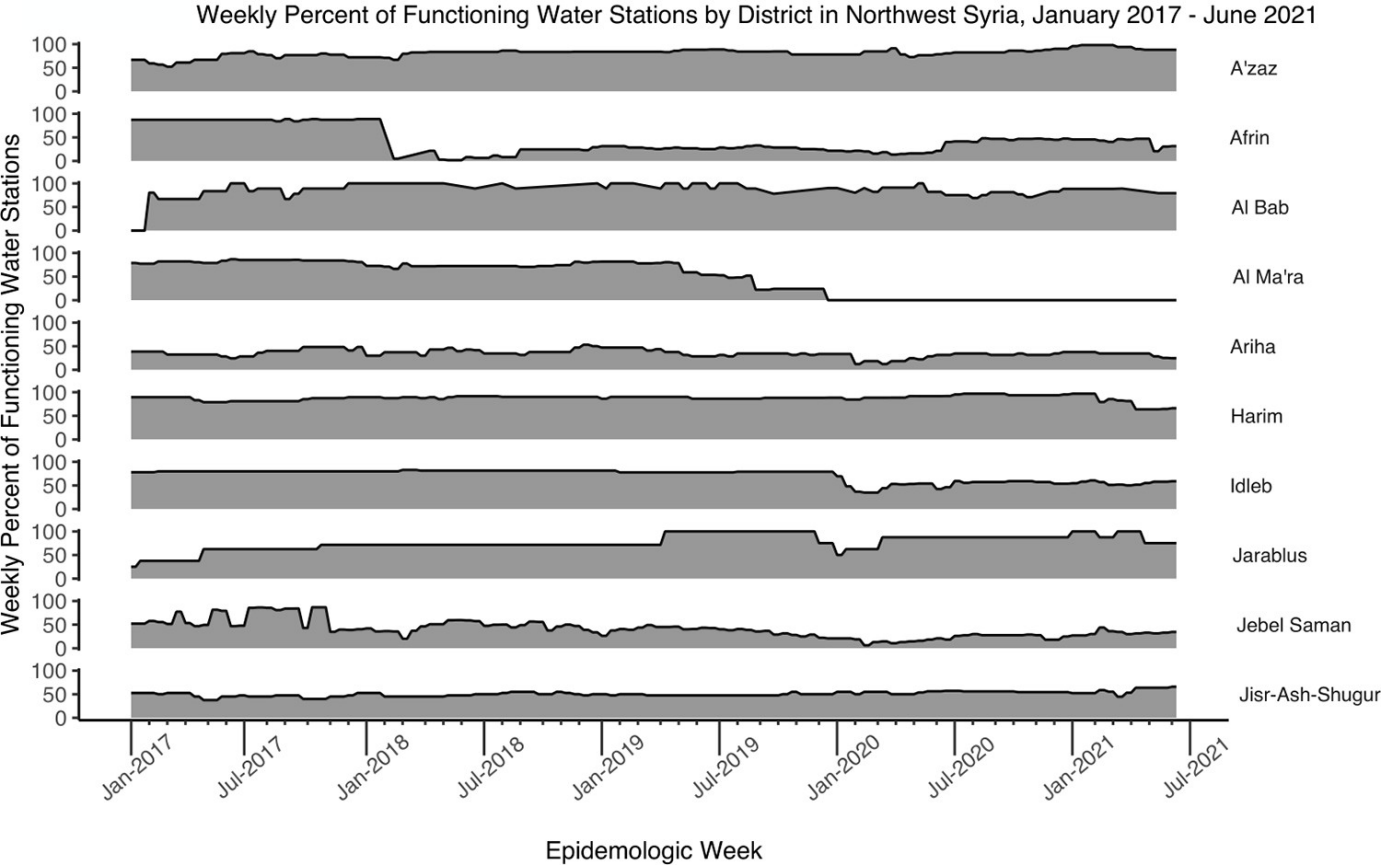
Weekly percent of functioning water stations by district in northwest Syria, January 2017- June 2021.

**Fig 7:**
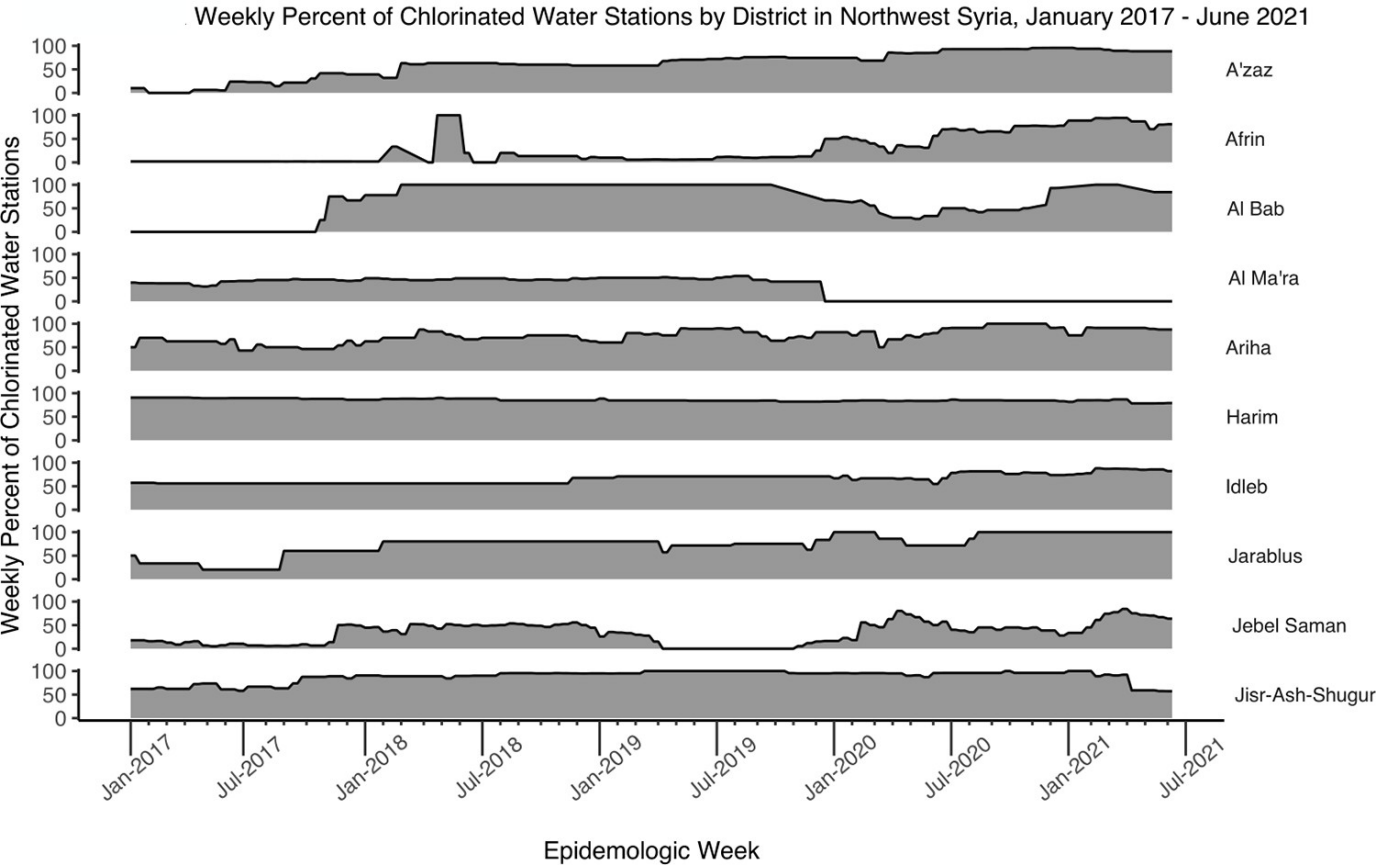
Weekly percent of chlorinated water stations by district in northwest Syria, January 2017 –June 2021.

The total number of water stations in the study areas gradually increased from 435 stations in January 2017 to 504 in December 2019 and then dropped to 367 in January 2020 (Fig 5). By the end of the study period, the number of water stations had increased to 588 in June 2021. Districts A’zaz, Al Bab, Harim, and Idleb had the highest number and most consistent proportions of functioning water stations, while Afrin, Ariha, and Jebel Saman had the lowest proportions of functioning water stations throughout the study period (Fig 6). Many of the districts, including Al Ma’ra, Ariha, Idleb, Jarablus, and Jebel Saman, had a drop in the proportion of functioning water stations in January 2020. Water stations had, in contrast, a low proportion of chlorination in the beginning of the time period, including water stations in A’zaz, Afrin, Al Bab, Jarablus, and Jebel Saman. Harim and Jisr-Ash-Shugur consistently had the highest proportions of chlorinated water stations during the study period, while Afrin, Al Ma’ra, and Jebel Saman had the lowest (Fig 7).

### Multivariable analyses

All negative binomial regression models provided a better fit than the Poisson regression models (p-value < 0.001); thus, we present results from the negative binomial models.

#### Functionality of water stations

Within the 10 districts in northwest Syria, as the quintile of functioning water stations increased, the incidences of AOD, ABD, AJS and STF decreased (Table 6). The rate ratios comparing quintiles two, three, four, and five to quintile one showed that the effect of functioning water stations on the incidences of each waterborne disease was increasingly protective. For example, the rate ratios for AOD comparing quintile two to quintile one (RR = 1.04, 95% CI = 0.92, 1.16) and quintile five to quintile one (RR = 0.55, 95% CI = 0.42, 0.72) decreased by 47%. Using the same comparison, the rate ratio for ABD decreased from 0.74 (95% CI = 0.57, 0.96) to 0.21 (95% CI = 0.11, 0.39), a 72% difference. The difference in the rate ratios for AJS from 1.25 (95% CI = 1.06, 1.49) to 0.55 (95% CI = 0.37, 0.83) was 56%. Finally, the rate ratio of STF decreased from 0.57 (0.47, 0.68) to 0.08 (0.05, 0.13), an 86% difference. Most of the rate ratio estimates calculated were statistically significant, with 95% confidence intervals, excluding 1.0. The interaction terms between percent of functioning and chlorinated water stations were statistically significant (p-value < 0.05) for each waterborne disease.

**Table 6:** Association between percent of functioning and chlorinated water stations and incidences of each of the four suspected waterborne diseases, northwest Syria, January 2017 –June 2021.

Exposure	No. Weeks (%)	AOD^c^ RR (95% CI)	ABD^c^ RR (95% CI)	AJS^c^ RR (95% CI)	STF^c^ RR (95% CI)
Percent of Functioning Water Stations^a^					
0 to <34.5^d^	424 (0.20)	1.00	1.00	1.00	1.00
34.5 to <52.5	434 (0.20)	1.04 (0.92, 1.16)	0.74 (0.57, 0.96)*	1.25 (1.06, 1.49)	0.57 (0.47, 0.68)*
52.5 to <77.4	413 (0.20)	0.74 (0.62, 0.89)*	0.26 (0.17, 0.39)*	0.92 (0.70, 1.20)	0.24 (0.18, 0.31)*
77.4 to <86	439 (0.20)	0.62 (0.48, 0.79)*	0.21 (0.12, 0.36)*	0.48 (0.33, 0.69)*	0.12 (0.08, 0.17)*
86 to 100	437 (0.20)	0.55 (0.42, 0.72)*	0.21 (0.11, 0.39)*	0.55 (0.37, 0.83)*	0.08 (0.05, 0.13)*
Percent of Chlorinated Water Stations^b^					
0 to <33.3^d^	437 (0.20)	1.00	1.00	1.00	1.00
33.3 to <55.8	356 (0.16)	0.84 (0.71, 0.99)*	0.44 (0.31, 0.64)*	1.00 (0.77, 1.28)	0.84 (0.65, 1.10)
55.8 to <75	513 (0.24)	0.71 (0.56, 0.89)*	0.44 (0.26, 0.73)*	0.82 (0.58, 1.17)	0.73 (0.5, 1.06)
75 to <88.2	445 (0.20)	0.47 (0.35, 0.63)*	0.30 (0.16, 0.58)*	0.87 (0.56, 1.35)	0.55 (0.35, 0.88)*
88.2 to 100	426 (0.20)	0.39 (0.28, 0.55)*	0.27 (0.13, 0.57)*	0.84 (0.50, 1.40)	0.41 (0.24, 0.71)*

^a^Four separate negative binomial regression models with exposure as percent of functioning water stations adjusted for district and week and an offset term of total population.

^b^Four separate negative binomial regression models with exposure as percent of chlorinated water stations adjusted for district and week and an offset term of total population.

^c^Acute Other Diarrhea (AOD) and Acute Bloody Diarrhea (ABD) were not lagged. Acute Jaundice Syndrome (AJS) was lagged by six weeks and Severe Typhoid Fever (STF) was lagged by two weeks.

^d^Reference category

*Risk ratio estimates that are statistically significant (i.e., 95% CI do not cross the null)

#### Chlorination of water stations

As the quintile of chlorinated water stations increased; the incidence of each waterborne disease decreased. The rate ratios of AOD comparing quintile two to quintile one (RR = 0.84, 95% CI = 0.71, 0.99) and quintile five to quintile one (RR = 0.39, 95% CI = 0.28, 0.55) decreased by 54%. Using the same comparison, the rate ratio of ABD decreased from 0.44 (0.31, 0.64) to 0.27 (0.13, 0.57), a 39% difference. The difference in the rate ratios for AJS from 1.00 (95% CI = 0.77, 1.28) to 0.84 (95% CI = 0.5, 1.4) was 16%. Finally, the rate ratio of STF decreased from 0.84 (0.65, 1.10) to 0.41 (95% CI = 0.24, 0.71), a 51% difference. Most of the rate ratio estimates were also statistically significant for each waterborne disease, except for AJS. The interaction term between percent of functioning and chlorinated water stations were statistically significant (p-value < 0.05) for each waterborne disease. (Table 6).

### Dose response analysis

Of the thirty dose-response models examined, only ten converged. Eight of these ten models were further adjusted for non-normality/heterogeneity through optimal Box-Cox transformations (lambda = 0.5). Dose-response results for convergent findings are presented in Table 7. The only models to converge were those for acute other diarrhea (AOD) and percentage of chlorinated water stations, and acute jaundice syndrome and functioning water stations (with a six-week lag period). All five cross-sections (including total cases, age group 0–4 years, age group > = 5 years, males only, and females only) were significant for these two diseases.

**Table 7:** Dose-response models for convergent findings.

Disease	Dose	Response	ED50 est	Std Error	t-value	p-value	Residual SE	Lower CI	Upper CI
Diarrhea	Chlorination	Total	13.07	2.35	5.56	< 0.000	20.8	8.46	17.69
Diarrhea	Chlorination	Age 0–4	11.53	2.07	5.58	< 0.000	15.81	7.48	15.59
Diarrhea	Chlorination	Age 5	16.52	3.6	4.58	< 0.000	252.8	9.46	23.59
Diarrhea	Chlorination	Male	12.25	2.15	5.67	< 0.000	14.62	8.02	16.48
Diarrhea	Chlorination	Female	13.93	2.58	5.4	< 0.000	14.71	8.87	18.99
Jaundice	Functionality	Total	24.15	1.23	19.62	< 0.000	5.31	21.73	26.56
Jaundice	Functionality	Age 0–4	24.19	1.31	18.51	< 0.000	3.71	21.63	26.76
Jaundice	Functionality	Age 5	24.05	1.23	19.59	< 0.000	3.99	21.65	26.46
Jaundice	Functionality	Male	24.29	1.18	20.66	< 0.000	3.82	21.98	26.59
Jaundice	Functionality	Female	23.78	1.71	13.91	< 0.000	18.17	20.43	27.13

*All models are 3 parameter log-logistic with lower limit at 0, diarrhea denoted Acute Other Diarrhea, jaundice denoted Acute Jaundice Syndrome (AJS)

For acute other diarrhea, the ED50 coefficients suggest that a fifty percent reduction in the disease for half of the population could theoretically be achieved by increasing the percentage of chlorinated stations by 13.1% (total cases), 11.5% (0–4 age group), 12.5% (males only), and 13.9% (females only). The ED50 coefficient of 16.3% for the 5 years and older age group is larger than the others and contains larger standard errors, a smaller t-value, and a much wider confidence interval than the other categories, possibly because this represents a wide age group ranging from young children to the elderly.

For acute jaundice syndrome, the ED50 coefficients suggest that a fifty percent reduction in the disease for half of the population could also theoretically be obtained by increasing the percentage of functioning stations by 24.2% (total cases), 24.2% (0–4 age group), 24.1 (5 years and older), and 24.3% (males only). Notably, the 23.8% coefficient for females only was accompanied by larger standard errors, a smaller t-value, and wider confidence intervals than the other categories.

## IV. Discussion

Our results link the rate of waterborne diseases to water station functionality and chlorination in northwest Syria where deliberate attacks on critical civilian infrastructure have been frequent. Increasing the percentage of functioning and chlorinated water stations was associated with a reduced risk of waterborne disease in high-conflict areas of northwest Syria between January 1, 2017, and June 30, 2021. Chlorination can deeply impact diarrheal disease in settings where the population generally has access to running water. But when running water is not consistently available, as for much of Syria’s conflict affected communities, water station functionality by itself may be an even more important variable. In demonstrating the associations between the maintenance of water stations in northwest Syria and preventing infectious diseases, we hope to advocate for better support for WASH programs in conflict settings.

We found that, compared to areas with lower quintiles of functioning and chlorinated water stations, the rates of all four waterborne diseases examined (acute other diarrhea, acute bloody diarrhea, severe typhoid fever, and acute jaundice syndrome) were lower in areas that had higher quintiles of functioning and chlorinated water stations. The presence of functioning water stations may have had a stronger association with lower rates of waterborne diseases than exposure to chlorinated water stations. This finding could be explained by noting that in conflict settings where all water is so restricted, access to running water, whether or not it is chlorinated, may be of primary importance. We did not have data on other methods of water disinfection that the population of interest might have had access to, such as chlorination or iodination tablets or boiling water. Higher quintiles of functioning water stations had the greatest impact on reducing the incidences of ABD and STF, while higher quintiles of chlorinated water stations had the greatest impact on reducing the incidences of AOD and STF.

The annual number of cases of waterborne disease in the study area gradually increased from 360,000 in 2017 to 440,000 in 2020. This might be due to multiple factors related to conflict and war, including a decrease in access to healthcare, sanitation, and hygiene, as well as an increase in displacement and overcrowding in refugee camps [5, 17]. Acute Other Diarrhea accounted for the largest number of cases of waterborne disease. Given that viral diarrhea, such as that caused by rotavirus and adenovirus, is one of the leading causes of morbidity and mortality globally, this finding is consistent with global data on diarrheal disease mortality [41]. Additionally, we offer further evidence that large impacts can be made to the incidence of diarrheal disease with relatively small investments in chlorination practices.

During the study period, districts varied by the number of water stations, that were present, functional and/or chlorinated, and by the number of waterborne disease cases. The total number of water stations in northwest Syria decreased dramatically after January 2020. This drop may have been related to the government’s attacks and occupation of part of northwest Syria in that time [42]. Many of the districts also saw a drop in the proportion of functioning water stations after January 2020. Many districts had inconsistency in the number of water stations that were functioning or chlorinated, including Afrin, Al Ma’ra, Ariha, and Jebel Saman. This was due both to water infrastructures being destroyed during bombing and airstrikes and the challenges of managing civil infrastructure in humanitarian emergencies [15, 18]. We were not able to identify which water stations were actively destroyed in the conflict, nor if districts with more dysfunctional water stations also faced more general violence, nor if other ecological factors could have influenced the findings. As such, this study did not test the relationship between direct attacks on water stations or WASH systems and waterborne diseases. However, our data suggest that when water stations are destroyed, people will get more diarrhea, jaundice and other waterborne illnesses.

Results of the dose response models suggest that reductions could be made in the rate of acute other diarrhea and acute jaundice syndrome by increasing the percentage of chlorinated water stations by roughly 10–15% and increasing the number of functioning water stations by roughly 24%, respectively. However, the picture is complicated because it is difficult to ascertain which populations are served by which water stations, accurate tracking of disease and strategic military targeting of water stations, and variation of effect by age and gender, among other factors. We stratified our results by age with the hypothesis that children may be more impacted by drinking water contamination than adults because they drink more fluid per pound of body weight than adults, young children’s immune systems are not yet fully developed, and maybe be more susceptible to contaminants and diseases that affect learning and motor skills [43]. While we found no significant differences between, more granular studies may be required. While a recent systematic review concludes suggests that gender does not significantly influence waterborne disease incidence it does impact water access as well as sanitation and hygiene needs [44]. We included gender in our sub-analysis to ensure we were not missing any gendered impacts of clean water and found no significant differences.

We also note that, consistent with long standing research, chlorination has a differentiated effect on the inactivation of different bacteria and viruses. Thus, at the standard levels of chlorination where ACU determines that water is adequately chlorinated, there may be different infectious capacities of different water borne diseases [45]. A recent outbreak of cholera in north east Syria has been linked to the deterioration and destruction of water treatment plants as well as multiple other factors including “overexploitation of groundwater, climate change, drought, and environmental contamination related to the conflict” [46, 47]. Our results underscore many calls for better water station functionality and chlorination, as well as protection of this critical infrastructure from attacks, to prevent such outbreaks across Syria and other conflict regions.

### Strengths and limitations

Our study had several important limitations. Because this analysis used population level data (i.e., was ecologic in nature), we cannot extrapolate our findings to the individual level. Moreover, the populations accessible to surveillance for our disease outcomes and water stations may not be representative of the entire population, because the ACU can access only regions that are outside of government control and that are at least safe enough to report from; as a result, we cannot generalize our findings to all of Syria or to other countries in conflict. Internal validity may be limited because of complications with data collection during a conflict, including physical, political, and safety limitations. However, most regions in northwest Syria have maintained high levels of completeness and timeliness of surveillance reports (estimated at greater than 95% by ACU representatives) [22]. In addition, while we calculated incidence rates based on HNAP population estimates, these values may be inaccurate due to the data collection methods used, which rely on surveys, interviews, and information sharing by local organizations, or inaccurate due to unmeasured population movements [48]. The population sizes of different regions are constantly changing during the conflict, due to migration within and outside of Syria, we could not control for population movements in our analysis. We also could not assess access to other drinking water or water disinfection practices that were not reported by ACU. For example, while water is rationed all over the country, some communities receive water via trucking (both as charity and private pay) or via other sources such as wells, rivers and springs that depend on electric water pumps [49–51]. ACU reports that 63% of the entire population uses water from the water station network(75% of communities and 42% of IDP camp populations), but in the future, sensitivity analysis may help solidify the analysis and provide more granularity, as published estimates are scant [20]. AJS had very wide confidence intervals and some were not statistically significant. This might be due to decreasing our sample size (number of weeks) for AJS because AJS was lagged by six weeks to account for incubation period. We ran Wilcoxon Rank Sum Tests to determine if there was a significant difference between male and female cases and between cases <5 years of age and cases **≥**5 years of age and to assess if there was a need to stratify our models by gender and age. Although we found significant differences in the rate of waterborne diseases between male and female cases (except for AOD) and between cases <5 and **≥**5 years of age (except for AJS), we did not stratify by gender and age and present our results as pooled estimates rather than stratified estimates because of resource constraints. More granular analysis would be an important next step. Although there are several limitations due to our study being in a conflict setting, ACU is the primary organization collecting disease surveillance and WASH data in non-government-controlled regions in Syria and the data we used are the most complete and timely data available.

### Recommendations

Our results underscore the critical importance of access to disinfected water and its effect in reducing enteric disease during conflict and war. The Syrian government and humanitarian as well as development organizations must prioritize expanding access to clean water by rebuilding damaged water infrastructure and supporting sustainable WASH-related projects. In addition to expanding access to WASH practices, the UN and national governments should hold all parties involved in the conflict accountable for the targeting of water infrastructure, which violates international humanitarian law and causes significant morbidity and mortality [52]. Despite statements from the UN and the International Criminal Court addressing the targeting of civilian infrastructure as a potential violation of international humanitarian law, airstrikes on water sources by all parties have been a common occurrence throughout the war [15, 18]. There are several opportunities for reparations and accountability: the UN could develop systems for documenting attacks on water sources (similar to the WHO SSA for attacks on healthcare) [53, 54]. The International Criminal Court or other accountability mechanisms could prioritize accountability for civilian infrastructure attacks and funding could be aligned more closely with the needs of vulnerable and conflict-affected communities suffering from these preventable diseases.

The results of this study allow stakeholders to have a better understanding of the effects of water station functionality and chlorination in conflict settings and thus prioritize improvement and expansion of WASH activities to countries facing conflict and war. Future work should continue to use and improve standardized conflict data collection methods. We hope this emphasizes the importance of collecting data, which requires well-contextualized and better funded support for population and movement data, disease surveillance and WASH programs in humanitarian settings.
